# Supplementary material for: Dietary supplementations to mitigate the cardiopulmonary effects of air pollution toxicity: A systematic review of clinical trials
Source: PLoS One. 2024 Jun 13;19(6):e0304402. doi: 10.1371/journal.pone.0304402 (PMC11175466; doi:10.1371/journal.pone.0304402)
Supplement: S1 Table — (DOCX) [file pone.0304402.s002.docx]

**Supplementary table 1. Search keywords and queries**

((((((((((((((((((((((((((((Vitamin C[Title/Abstract]) OR (vitamin E[Title/Abstract])) OR (carotine[Title/Abstract])) OR (Sulforaphane[Title/Abstract])) OR (Vitamin A[Title/Abstract])) OR (carotenoid*[Title/Abstract])) OR (Vitamin D[Title/Abstract])) OR (Omega[Title/Abstract])) OR (polyunsaturat*[Title/Abstract])) OR (fatty acid[Title/Abstract])) OR (PUFA[Title/Abstract])) OR (omega 3[Title/Abstract])) OR (omega-3[Title/Abstract])) OR (fish oil[Title/Abstract])) OR (Soy oil[Title/Abstract])) OR (Olive oil[Title/Abstract])) OR (Glucoraphanin[Title/Abstract])) OR (acetylcysteine[Title/Abstract])) OR (carotine[Title/Abstract])) OR (Vitamin[Title/Abstract])) OR (Dietary Supplement*[Title/Abstract])) OR (Dietary Supplements[MeSH Terms])) OR (Micronutrients[MeSH Terms])) OR (Micronutrient*[MeSH Terms])) OR (Trace Elements[MeSH Terms])) OR (Trace Element*[Title/Abstract])) AND (((((((((((((((((((((((((((((((((((((((((air pollution[MeSH Terms]) OR (air pollution[Title/Abstract]))) OR (Air Pollutants[MeSH Terms])) OR (Air Pollutant[Title/Abstract])) OR (air pollution indicator[Title/Abstract])) OR (Particulate Matter[MeSH Terms])) OR (Particulate Matter[Title/Abstract])) OR (nitrogen dioxide[MeSH Terms])) OR (nitrogen dioxide[Title/Abstract])) OR (Carbon Monoxide[MeSH Terms])) OR (Carbon Monoxide[Title/Abstract])) OR (Sulfur Dioxide[Title/Abstract])) OR (Sulfur Dioxide[MeSH Terms])) OR (Ozone[MeSH Terms])) OR (Ozone[Title/Abstract])) OR (acetylene[Title/Abstract])) OR (acetylene[MeSH Terms])) OR (benzene[Title/Abstract])) OR (butadiene[Title/Abstract])) OR (butadiene[MeSH Terms])) OR (ethane[MeSH Terms])) OR (ethane[Title/Abstract])) OR (ethylbenzene[Title/Abstract])) OR (ethylbenzene[MeSH Terms])) OR (ethylene[MeSH Terms])) OR (ethylene[Title/Abstract])) OR (toluene[Title/Abstract])) OR (toluene[MeSH Terms])) OR (xylene[MeSH Terms])) OR (xylene[Title/Abstract])) OR (polycyclic aromatic hydrocarbon[Title/Abstract])) OR (polycyclic aromatic hydrocarbon[MeSH Terms])) OR (black carbon[Title/Abstract])) OR (volatile organic compound[Title/Abstract])) OR (exhaust*[Title/Abstract])) OR (acetylene*[Title/Abstract] OR benzene*[Title/Abstract] OR butadiene*[Title/Abstract] OR co[Title/Abstract] OR carbonmonoxide*[Title/Abstract])) OR (Coarse[Title/Abstract] OR dust[Title/Abstract] OR ethane[Title/Abstract] OR ethylbenzene*[Title/Abstract] OR ethylene*[Title/Abstract] OR ethene*[Title/Abstract] OR particle*[Title/Abstract] OR nitro*[Title/Abstract] OR pm1[Title/Abstract] OR 'pm2 5'[Title/Abstract] OR pm10[Title/Abstract] OR 'pm 1'[Title/Abstract] OR 'pm 2 5'[Title/Abstract] OR 'pm 10'[Title/Abstract] OR soot[Title/Abstract] OR toluene*[Title/Abstract] OR xylene*[Title/Abstract] OR ufp*[Title/Abstract])) OR (PM2.5[Title/Abstract])) OR (Diesel exhaust particle[Title/Abstract])) AND ((clinicaltrial[Filter] OR controlledclinicaltrial[Filter] OR randomizedcontrolledtrial[Filter]) AND (humans[Filter]) AND (2000/1/1:2023/1/29[pdat]) AND (english[Filter])) AND ((clinicaltrial[Filter] OR controlledclinicaltrial[Filter] OR randomizedcontrolledtrial[Filter]) AND (humans[Filter]) AND (2000/1/1:2023/1/29[pdat]) AND (english[Filter]))
