# Supplementary material for: Dietary supplementations to mitigate the cardiopulmonary effects of air pollution toxicity: A systematic review of clinical trials
Source: PLoS One. 2024 Jun 13;19(6):e0304402. doi: 10.1371/journal.pone.0304402 (PMC11175466; doi:10.1371/journal.pone.0304402)
Supplement: Attachment — Submitted filename: PLOS ONE_020824.docx [file pone.0304402.s003.docx]

**Dietary Supplementations to Mitigate the Cardiopulmonary Effects of Air Pollution Toxicity: A Systematic Review of Clinical Trials** Ilaghi *et al*. 2024

This paper attempts to inform the literature of the beneficial effects of certain dietary supplements in populations and settings of poor air quality/air pollutant exposure. Authors utilized a systematic review approach to assess the impacts of particulate matter and ozone exposure on cardiovascular and pulmonary functioning and the potential for dietary supplements to mitigate these effects. Results of this systemic review were inconclusive in regard to the overall benefit of dietary supplementation in these exposure scenarios. The inconclusive results may be due to the wide array of studies included in this review, as discussed in the comments below. In addition to stylistic and organizational shortfalls of the paper, there are deficiencies in the background and rationale provided as well as fundamental flaws in the review.

Comments:

1. This review is focused on the *cardio*pulmonary effects of air pollution. However, the introduction only discusses the basic mechanisms through which particulates and other pollutants contribute to pulmonary detriments with no mention of the cardiovascular effects. Discussion of how pollutant exposure contributes to cardiovascular disease and alterations in cardiovascular functioning (specifically those discussed in the review) should be included.
2. Authors do not provide context as to why these outcome measures were chosen for evaluation and how they are illustrative cardiopulmonary dysfunction as mentioned above.
3. Authors should also elaborate further as to how dietary supplements, drawing on epidemiological and mechanistic research, may mitigate the deleterious effects of pollutants.
4. Check for proper use of defined abbreviations throughout.
5. There are multiple B vitamins, authors should be more specific about which B vitamin or combination of them is being evaluated in each study.
6. Authors should mention dosages and duration of both supplementation and pollutant exposure within the text.
7. The discussion provides description of metabolic and cellular changes resulting from dietary supplementation and pollution but there is very little of the results into this discussion.
8. The inconclusiveness of these findings does not inform the literature. Inconclusiveness is likely related to the wide array of outcomes measures and the number of dietary supplement interventions assessed in the review. Due to the variety in the studies included, in the review, it is difficult to make comparisons or conclusions on specific dietary supplements or parameters of interest.
